# Correlation between multifidus muscle atrophy, spinopelvic parameters, and severity of deformity in patients with adult degenerative scoliosis: the parallelogram effect of LMA on the diagonal through the apical vertebra

**DOI:** 10.1186/s13018-019-1323-6

**Published:** 2019-08-28

**Authors:** Xiang-Yao Sun, Chao Kong, Tong-Tong Zhang, Shi-Bao Lu, Wei Wang, Si-Yuan Sun, Ma-Chao Guo, Jun-Zhe Ding

**Affiliations:** 1 Department of Orthopaedics, Xuanwu Hospital Capital Medical University, Beijing, 100053 China; 2 National Clinical Research Center for Geriatric Diseases, Beijing, China; 3 Capital Medical University, Beijing, China; 4 Department of Orthopaedics, ChuiYangLiu Hospital affiliated to Tsinghua University, Beijing, China

**Keywords:** Adult degenerative scoliosis, Lumbar multifidus muscle atrophy, Spinopelvic parameters, Sagittal imbalance, Correlation

## Abstract

**Background:**

There were several reports describing the biomechanics and microstructure of multifidus muscles in patients with lumbar disc herniation. However, correlations between lumbar multifidus muscle atrophy (LMA), spinopelvic parameters, and severity of adult degenerative scoliosis (ADS) have not been investigated. The study evaluated the impact of LMA and spinopelvic parameters on the severity of ADS.

**Methods:**

One hundred and thirty-two patients with ADS were retrospectively reviewed. Standing whole-spine X-ray was used to evaluate the coronal (coronal Cobb angle, CA; coronal vertical axis, CVA) and sagittal (sagittal vertical axis, SVA; thoracic kyphosis, TK; lumbar lordosis, LL; pelvic incidence, PI; pelvic tilt, PT; sacral slope, SS) parameters. LMA was evaluated on axial T2-weighted magnetic resonance imaging (MRI) at intervertebral levels above and below the vertebra at the apex of the scoliotic curve. Clinical symptoms were evaluated by the Oswestry Disability Index (ODI) and the Japanese Orthopaedic Association (JOA) score. Multiple linear regression was used to assess correlations between LMA, spinopelvic parameters, and severity of scoliosis.

**Results:**

LL and PT were negatively correlated with CA (*P <* 0.001); LL was positively correlated with SVA (*P* < 0.001). PI was positively correlated with CA (*P* < 0.001) and CVA (*P* < 0.001). PT (*P* < 0.001) and SS (*P* < 0.001) were negatively correlated with CVA. SS was negatively correlated with SVA (*P* < 0.001). Concave LMA at the upper or lower intervertebral level of the apical vertebra was positively correlated with CA (*P* ≤ 0.001); convex LMA at the upper or lower intervertebral level was negatively correlated with CA (*P* < 0.001). Convex LMA at the upper intervertebral level and concave LMA at the lower intervertebral level of the apical vertebra were negatively correlated with the SVA (*P* ≤ 0.001). At the upper intervertebral level, LMA on the concave side was positively correlated with CVA (*P* = 0.028); LMA on the convex side was negatively correlated with CVA (*P* = 0.012). PI was positively correlated with ODI (*P* < 0.001); PT (*P* < 0.001) and SS (*P* < 0.001) were negatively correlated with ODI. At the lower intervertebral level, LMA on the concave side was positively correlated with ODI (*P* = 0.038); LMA on the convex side was negatively correlated with ODI (*P* = 0.011). PI was positively correlated with JOA (*P* < 0.001); PT (*P* < 0.001) and SS (*P* < 0.001) were negatively correlated with JOA.

**Conclusions:**

Spinopelvic parameters are correlated with the severity of ADS. Asymmetric LMA at both upper and lower intervertebral levels of the apical vertebra is positively correlated with CA. LMA on the diagonal through the apical vertebra is very important to maintain sagittal imbalance via parallelogram effect. LMA at lower intervertebral levels of the apical vertebra may have a predictive effect on ODI. JOA score seems to be more correlated with spinopelvic parameters than LMA.

## Background

Adult degenerative lumbar scoliosis (ADS) is defined as spinal deformity with a coronal deviation of greater than 10° in a skeletally mature patient, especially older than 40 years, without a history of scoliosis in childhood or adolescence [1]. The prevalence of ADS rises with age, with estimates ranging from 6 to 68%; therefore, it is becoming a major public health concern as the global incidence is increasing with the aging population [2, 3].

There were several reports describing the biomechanics and microstructure of multifidus muscles in patients with lumbar disc herniation [4]. Results showed correlations between lumbar multifidus muscle atrophy (LMA) and chronic low back pain, disc degeneration, and radiculopathy [5, 6]. However, correlations between LMA, spinopelvic parameters, and severity of ADS have not been investigated. Yagi et al. [7] reported that the cross-sectional area (CSA) of the multifidus (MF) and psoas (PS) were significantly smaller in degenerative lumbar scoliosis (DLS) patients. However, their muscle CSA analysis only included the L5-S1 level, which could not reflect the characteristics of paravertebral muscles around the apex vertebra. Our study simplified the approach to assessing LMA by using Goutallier Classification system [8]; LMA on the convex and concave sides of the scoliotic curve was measured on axial T2-weighted MR images at intervertebral levels above and below the apical vertebras. All of them would make the results more applicable.

The purpose of this study was to evaluate the correlation between LMA, spinopelvic parameters, and the severity of ADS and identify variables that predict progression in ADS. To our knowledge, this has not been previously reported.

## Methods

### Selection criteria

Patients with ADS that attended our inpatient clinic during the period from January 2016 to December 2017 were eligible for study. Inclusion criteria were age > 40 years at the time of attendance, medical records containing anteroposterior and lateral X-ray radiographs of total spine and magnetic resonance imaging (MRI) of the lumbar spine, and Cobb angle of lumbar curve in the coronal plane > 10° on a standing posteroanterior film. Exclusion criteria were history of scoliosis in childhood or adolescence, history of spinal surgery, local infection, inflammation around the spine, history of severe spinal trauma, spinal tumor, and presence of other systemic diseases that can affect spinal alignment (e.g., muscular dystrophy, ankylosing spondylitis, Parkinson disease). All patients included in our study provided written informed consent. This study has been approved by the institutional review board following the Declaration of Helsinki principles.

### Evaluation of muscles in MRIs

**Fig. 1:**
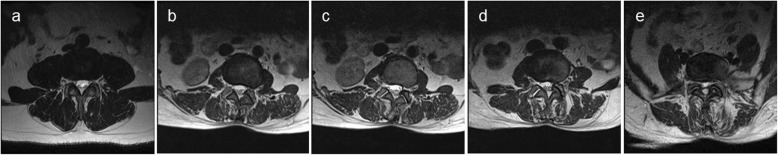
Goutallier grades (range, 0 to 4) on axial T2W1 MRI are represented by **a** to **e**: grade 0, normal muscle tissue (**a**); grade 1, fat streaks (**b**); grade 2, more muscle than fat (**c**); grade 3, equal amounts of fat and muscle tissue (**d**); and grade 4, more fat than muscle (**e**)

In this study, 1.5 imaging system (Magnetom Symphony; Siemens, Berlin, Germany) for MRIs was used, and three T2-weighted axial images at intervertebral levels above and below the apical vertebras were obtained. The slices were separated by a 0.1-mm gap and were 4 mm thick; multifidus muscles on the convex and concave sides of the scoliotic curve were analyzed from the center slice of each of the three T2-weighted axial images. Muscle atrophy was known to be related to increased fatty infiltration; therefore, the Goutallier Classification system [8] was used to quantify muscle fatty degeneration in the lumbar multifidus muscle (Fig. 1).

### Radiographic measurement and analysis

**Fig. 2:**
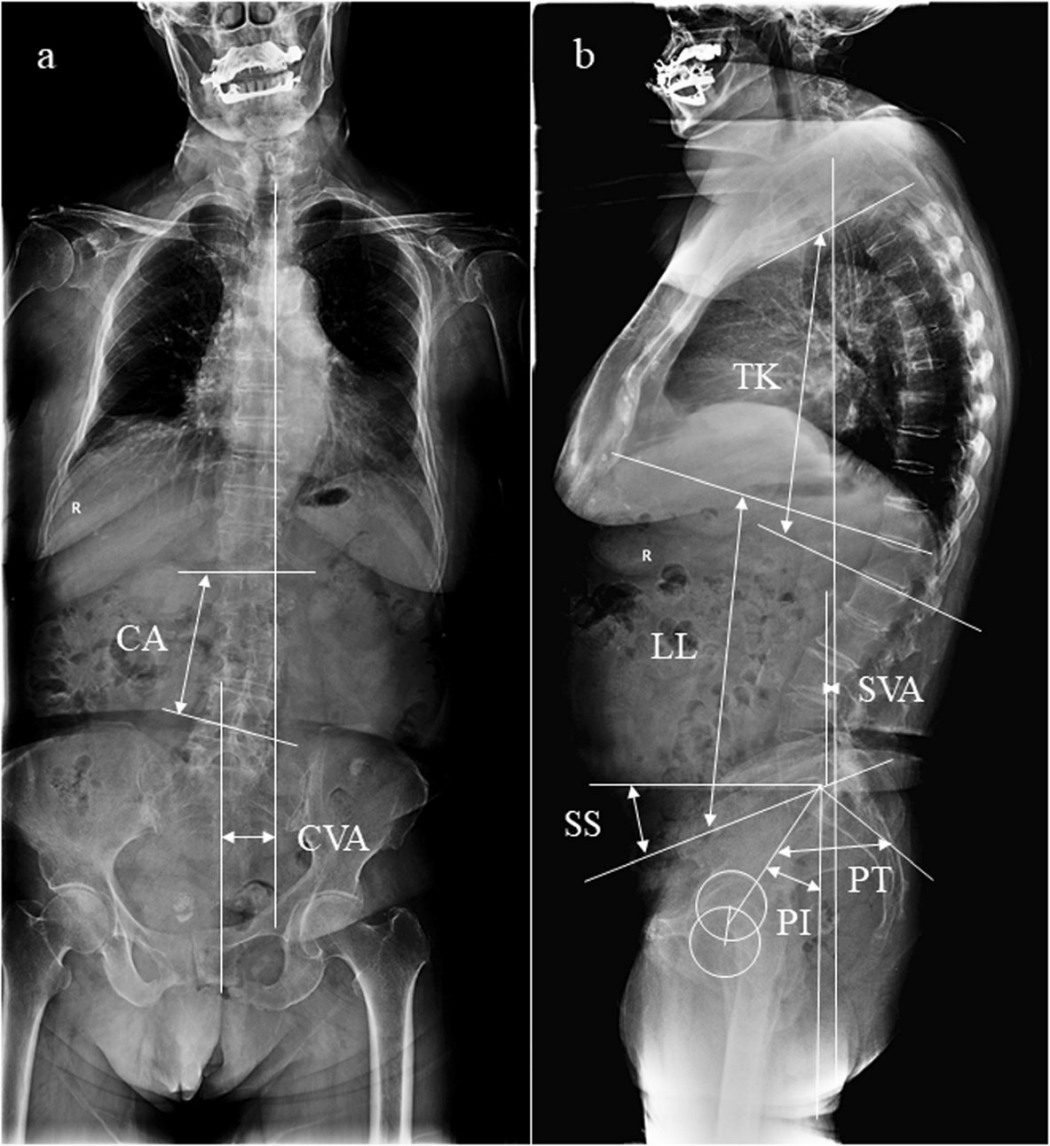
Radiological parameter measurement methods. **a** The coronal Cobb angle (CA) is measured from the superior end plate of the cephalad end vertebra and the inferior end plate of the caudal end vertebra on the coronal plane; the coronal vertical axis (CVA) is defined as the distance from a perpendicular line drawn from the superior end plate of S1 vertebral body to the C7 plumb line on the coronal plane. **b** The sagittal vertical axis (SVA) is defined as the distance from a perpendicular line drawn from the superior end plate of S1 vertebral body to the C7 plumb line on lateral radiographs; the thoracic kyphosis (TK) is measured from the upper end plate of T2 to the lower end plate of T12; the lumbar lordosis (LL) is measured from the upper end plate of T12 to the upper end plate of S1; the sacral slope (SS) is defined as the angle between the cranial sacral end plate and a horizontal line; the pelvic incidence (PI) is defined as the angle subtend by a line connecting the center of the femoral head to the center of the cephalad end plate of S1 and a perpendicular line from the upper end plate of S1; the pelvic tilt (PT) is measured as the angle between the vertical axis and the line through the midpoint of the sacral end plate to femoral heads axis

Standing whole-spine X-ray (Philips Digital Diagnost; Zhejiang Province, China) was used to evaluate the patients (Fig. 2). All radiologic parameters were measured twice at 1-month intervals by two researchers who were not involved in the patient encounters.

### Clinical assessment

Demographic data, including patients’ age and sex, were recorded. Clinical symptoms were evaluated by the Oswestry Disability Index (ODI) and the Japanese Orthopaedic Association (JOA) score. All of them were valid and rigorous functional measures used for assessing ADS.

### Statistical analysis

Statistical analyses were performed using Statistical Package for the Social Sciences version 17.0 software (SPSS, Inc., Chicago, IL). Non-contiguous data was presented as number or ratio, whereas continuous variables were reported as mean ± standard deviations (SD). Shapiro-Wilk test was used to test for normality. Wilcoxon rank-sum test was used to compare the differences in LMA between the concave and convex sides at intervertebral levels above and below the apical vertebras, or between upper intervertebral levels and lower intervertebral levels of the apical vertebras in concave or convex sides. Pearson’s correlation test was used to analyze the correlation between different parameters. Multiple linear regression was used to assess correlations between LMA, spinopelvic parameters, and severity of scoliosis. Statistical significance was set at *P* < 0.05.

## Results

### Patient demographics

This study included 132 ADS patients (42 males, 90 females) with a mean age of 61.5 ± 7.2 years (Table 1). Scoliotic curves typically had an apex at L3 (42.4%) and were convex to the left (58.3%). Mean CA was 25.4 ± 1.0°, TK was 31.2 ± 12.5°, LL was 29.7 ± 12.5°, PI was 53.4 ± 8.0°, PT was 29.2 ± 7.3°, and SS was 27.3 ± 7.5°. There was a wide range of severity of sagittal imbalance in these patients with a mean value of 8.9 ± 10.0 cm.

**Table 1:** Patient demographics

Number of cases	132
Gender (male/female)	42/90
Disease duration (months)	70.7 ± 24.2
Age (year)	61.5 ± 7.2
CA (°)	25.4 ± 1.0
TK (°)	31.2 ± 12.5
LL (°)	24.9 ± 21.6
PI (°)	53.4 ± 8.0
PT (°)	29.2 ± 7.3
SS (°)	23.5 ± 11.0
CVA (cm)	2.1 ± 1.0
SVA (cm)	8.9 ± 10.0
Apex of ADS	
L1	10 (7.6%)
L2	43 (32.6%)
L3	56 (42.4%)
L4	23 (17.4%)
Side of convex	
Left	77 (58.3%)
Right	55 (41.7%)

*CA* coronal Cobb angle, *TK* thoracic kyphosis, *LL* lumbar lordosis, *SS* sacral slope, *PI* pelvic incidence, *PT* pelvic tilt, *SVA* sagittal vertical axis, *ADS* adult degenerative scoliosis

### Comparison of LMA between different intervertebral levels or sides

**Fig. 3:**
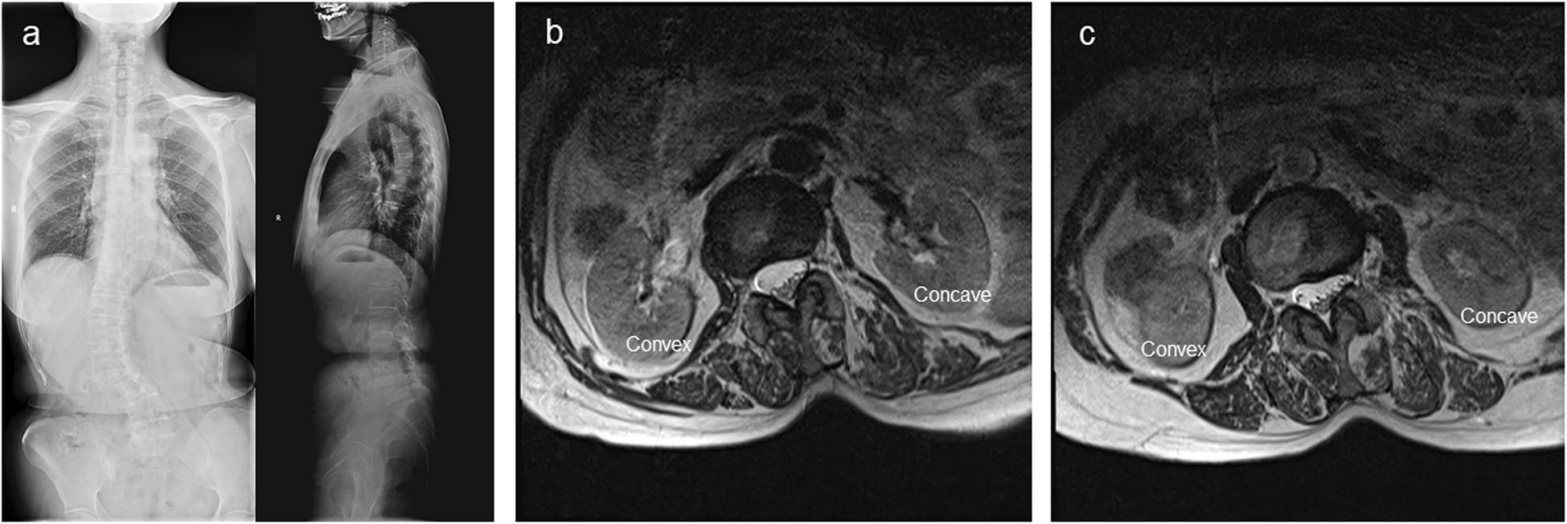
A 58-year-old female patient with adult degenerative scoliosis (ADS). **a** Standing anteroposterior (AP) and lateral radiographs. Coronal Cobb angle (CA), 35.6°; coronal vertical axis (CVA), − 1.6 cm; sagittal vertical axis (SVA), + 4.8 cm; thoracic kyphosis (TK), 9.2°; lumbar lordosis (LL), 28.1°; pelvic incidence (PI), 62.6°; pelvic tilt (PT), 35.2°; sacral slope (SS), 27.4°. **b** T2-weighted axial image at intervertebral levels above the apical vertebra (T12/L1): grade 1 LMA on the convex side and grade 4 LMA on the concave side. **c** T2-weighted axial images at the intervertebral level below the apical vertebra (L1/2): grade 2 LMA on the convex side and grade 3 LMA on the concave side

The Goutallier Classification system showed the following: at the upper intervertebral level, LMA (upper LMA, U-LMA) significantly increased on the concave side compared with the convex side (convex vs. concave, *Z* = − 7.616, *P* < 0.001); at the lower intervertebral level, LMA (lower LMA, L-LMA) similarly increased on the concave side compared with the convex side (convex vs. concave, *Z* = − 2.345, *P* = 0.019); on the concave side, no significant difference was found in between U-LMA and L-LMA (lower vs. upper, *Z* = − 0.093, *P* = 0.926); and on the convex side, U-LMA was significantly increased compared with L-LMA (lower vs. upper, *Z* = − 7.049, *P* < 0.001; Table 2; Fig. 3).

**Table 2:** Wilcoxon rank-sum test comparing LMA between different intervertebral levels or sides of the scoliosis

Variables	Goutallier grade	Concave side	Convex side	*Z* value (convex-concave)	*P* value
Upper intervertebral level	0	0	11	− 7.616	< 0.001
	1	5	6		
	2	17	49		
	3	66	44		
	4	44	22		
Lower intervertebral level	0	0	6	− 2.345	0.019
	1	6	5		
	2	16	11		
	3	66	33		
	4	44	77		
*Z* value (lower-upper)		− 0.093	− 7.049		
*P* value		0.926	< 0.001		

*LMA* lumbar multifidus muscle atrophy

### Correlation of spinopelvic parameters and LMA

Pearson correlation analysis showed a positive correlation between SS and LL (coefficient = 0.900, *P* < 0.001); LL and U-LMA on the concave side (coefficient = 0.212, *P* = 0.015); PI and LL (coefficient = 0.621, *P* < 0.001); PI and SS (coefficient = 0.758, *P* < 0.001); PI and L-LMA on the concave side (coefficient = 0.218, *P* = 0.012); PT and L-LMA on the concave side (coefficient = 0.442, *P* < 0.001); PT and L-LMA on the convex side (coefficient = 0.406, *P* < 0.001); U-LMA on the concave side and convex side (coefficient = 0.718, *P* < 0.001); U-LMA on the concave side and L-LMA on the concave side and on the convex side (coefficient = 0.385, *P* < 0.001; coefficient = 0.505, *P* < 0.001, respectively); U-LMA on the convex side and L-LMA on the concave side and the convex side (coefficient = 0.628, *P* < 0.001; coefficient = 0.502, *P* < 0.001, respectively); L-LMA on the concave side and L-LMA on the convex side (coefficient = 0.668, *P* < 0.001; Table 3). There were negative correlations between PT and LL and SS (coefficient = − 0.561, *P* < 0.001; coefficient = − 0.575, *P* < 0.001, respectively). Therefore, pelvic parameters and LMA in different positions were taken into multiple linear regression analysis.

**Table 3:** Pearson correlation analysis of spinopelvic parameters and concave or convex LMA at the upper or lower intervertebral level of the apical vertebra

Variables		TK	LL	SS	PI	PT	Upper intervertebral level LMA		Lower intervertebral level LMA	
							Concave side	Convex side	Concave side	Convex side
TK	Correlation	1.000	0.000	0.000	0.000	0.000	− 0.023	− 0.010	0.022	0.016
	*P* value		1.000	1.000	1.000	1.000	0.796	0.912	0.800	0.852
LL	Correlation		1.000	0.900	0.621	− 0.561	0.212	0.006	− 0.141	0.047
	*P* value		–	< 0.001	< 0.001	< 0.001	0.015	0.947	0.108	0.593
SS	Correlation			1.000	0.758	− 0.575	− 0.025	− 0.124	− 0.080	− 0.074
	*P* value			–	< 0.001	< 0.001	0.779	0.156	0.363	0.396
PI	Correlation				1.000	0.047	− 0.012	− 0.070	0.218	0.168
	*P* value				–	0.589	0.888	0.424	0.012	0.054
PT	Correlation					1.000	− 0.042	0.118	0.442	0.406
	*P* value					–	0.633	0.178	< 0.001	< 0.001
Upper intervertebral level LMA										
Concave side	Correlation						1.000	0.718	0.385	0.505
	*P* value						–	< 0.001	< 0.001	< 0.001
Convex side	Correlation							1.000	0.628	0.502
	*P* value							–	< 0.001	< 0.001
Lower intervertebral level LMA										
Concave side	Correlation								1.000	0.668
	*P* value								–	< 0.001
Convex side	Correlation									1.000
	*P* value									–

*LMA* lumbar multifidus muscle atrophy, *TK* thoracic kyphosis, *LL* lumbar lordosis, *SS* sacral slope, *PI* pelvic incidence, *PT* pelvic tilt

### Correlation of influencing factors and severity of ADS

Multiple linear regression (*R*^2^ = 0.705) of spinal pelvic parameters and CA showed there was a positive correlation between PI and CA (*B* = 1.519, *P* < 0.001); the correlations between PT (*B* = − 0.335, *P* < 0.001), LL (*B* = − 0.558, *P* < 0.001), and CA were negative (Table 4). On the concave side, both U-LMA (*B* = 4.266, *P* = 0.001) and L-LMA (*B* = 13.343, *P* < 0.001) were positively correlated with CA; on the convex side, both U-LMA (*B* = − 8.123, *P* < 0.001) and L-LMA (*B* = − 4.272, *P* < 0.001) were negatively correlated with CA; *R*^2^ value of this model was 0.500 (Table 4). Table 5, which summarizes the correlation between spinal pelvic parameters, L-LMA, or U-LMA on concave or convex side and SVA, shows that LL was positively correlated with SVA (*B* = 0.342, *P* < 0.001) and SS was negatively correlated with SVA (*B* = − 0.751, *P* < 0.001); because *R*^2^ value was 0.129, this model made little meaning; there were negative correlations between U-LMA on the convex side (*B* = − 8.123, *P* <  0.001), L-LMA (*B* = − 4.198, *P* = 0.001) on the concave side, and SVA (*R*^2^ = 0.319); *R*^2^ value of this model indicated a poor predictive power. Multiple linear regression analysis (*R*^2^ = 0.764; Table 6) of spinal pelvic parameters and CVA revealed that PI was positively correlated with CVA (*B* = 0.271, *P* < 0.001); PT (*B* = − 0.237, *P* < 0.001) and SS (*B* = − 0.182, *P* < 0.001) were negatively correlated with CVA. At the upper intervertebral level, LMA on the concave side was positively correlated with CVA (*B* = 0.364, *P* = 0.028); LMA on the convex side was negatively correlated with CVA (*B* = − 0.296, *P* = 0.012); considering the *R*^2^ value was 0.050, the meaning of this multiple linear regression model was limited (Table 6). Multiple linear regression (*R*^2^ = 0.680) of spinal pelvic parameters and ODI showed PI was positively correlated with ODI (*B* = 0.407, *P* < 0.001); PT (*B* = − 0.229, *P* < 0.001) and SS (*B* = − 0.466, *P* < 0.001) were negatively correlated with ODI (Table 7). At the lower intervertebral level, LMA on the concave side was positively correlated with ODI (*B* = 0.735, *P* = 0.038); LMA on the convex side was negatively correlated with ODI (*B* = − 0.668, *P* = 0.011); however, this multiple linear regression model made little sense (*R*^2^ = 0.051; Table 7). PI was positively correlated with JOA (*B* = 0.192, *P* < 0.001); PT (*B* = − 0.119, *P* < 0.001) and SS (*B* = − 0.213, *P* < 0.001) were negatively correlated with JOA; *R*^2^ value of this model was 0.687 (Table 8). Duration of disease was positively correlated with SVA (*B* = 0.138, *P* < 0.001, *R*^2^ = 0.111) and negatively correlated with CA (*B* = − 0.086, *P* = 0.017, *R*^2^ = 0.043); no significant correlation was found between duration of disease and CVA (*P* = 0.838), ODI (*P* = 0.352), and JOA (*P* = 0.121). Considering the *R*^2^ value of this model was very low, duration of disease was not a good predictor of the severity of ADS.

**Table 4:** Multiple linear regression analysis of influencing factors and CA

Influencing factors	Variables	*B* value	Standard error	*t* value	*P* value	*R*^2^ value
Spinopelvic parameters	LL	− 0.335	0.043	− 7.749	< 0.001	0.705
	PI	1.519	0.093	15.781	< 0.001	
	PT	− 0.558	0.100	− 5.569	< 0.001	
	Constant	− 31.079	3.753	− 8.281	< 0.001	
Concave or convex LMA at upper or lower intervertebral level of the apical vertebra	Upper intervertebral level LMA					0.500
	Concave	4.226	1.254	3.369	0.001	–
	Convex	− 8.123	1.000	− 8.122	< 0.001	
	Lower intervertebral level LMA					–
	Concave	13.343	1.253	10.645	< 0.001	–
	Convex	− 4.272	0.850	− 5.024	< 0.001	
	Constant	4.522	3.573	1.266	0.208	–

*CA* coronary Cobb angle, *LL* lumbar lordosis, *PI* pelvic incidence, *PT* pelvic tilt, *LMA* lumbar multifidus atrophy

**Table 5:** Multiple linear regression analysis of influencing factors and SVA

Influencing factors	Variables	*B* value	Standard error	*t* value	*P* value	*R*^2^ value
Spinopelvic parameters	LL	0.342	0.088	3.904	< 0.001	0.129
	SS	− 0.751	0.172	− 4.362	< 0.001	
	Constant	18.093	2.434	7.433	< 0.001	
Concave or convex LMA at the upper or lower intervertebral level of the apical vertebra	Upper intervertebral level LMA					0.319
	Convex	− 8.123	1.000	7.615	< 0.001	–
	Lower intervertebral level LMA					–
	Concave	− 4.198	1.184	− 3.545	0.001	–
	Constant	5.941	2.974	1.997	0.048	–

*LL* lumbar lordosis, *SS* sacral slope, *SVA* sagittal vertical axis, *LMA* lumbar multifidus atrophy

**Table 6:** Multiple linear regression analysis of influencing factors and CVA

Influencing factors	Variables	*B* value	Standard error	*t* value	*P* value	*R*^2^ value
Spinopelvic parameters	PI	0.271	0.20	13.550	< 0.001	0.764
	PT	− 0.237	0.018	− 13.549	< 0.001	
	SS	− 0.182	0.018	− 10.156	< 0.001	
	Constant	− 1.159	0.361	− 3.215	0.002	
Concave or convex LMA at the upper or lower intervertebral level of the apical vertebra	Upper intervertebral level LMA					0.050
	Concave	0.364	0.163	2.229	0.028	–
	Convex	− 0.296	0.116	− 2.545	0.012	–
	Constant	1.700	0.375	4.535	< 0.001	–

*PI* pelvic incidence, *PT* pelvic tilt, *SS* sacral slope, *CVA* coronal vertical axis, *LMA* lumbar multifidus atrophy

**Table 7:** Multiple linear regression analysis of influencing factors and ODI

Influencing factors	Variables	*B* value	Standard error	*t* value	*P* value	*R*^2^ value
Spinopelvic parameters	PI	0.407	0.055	7.462	< 0.001	0.680
	PT	− 0.229	0.048	− 4.788	< 0.001	
	SS	− 0.466	0.049	− 9.556	< 0.001	
	Constant	57.972	0.984	58.886	< 0.001	
Concave or convex LMA at the upper or lower intervertebral level of the apical vertebra	Lower intervertebral level LMA					0.051
	Concave	0.735	0.350	2.099	0.038	
	Convex	− 0.668	0.258	− 2.586	0.011	
	Constant	61.984	0.848	73.134	< 0.001	

*PI* pelvic incidence, *PT* pelvic tilt, *SS* sacral slope, *ODI* Oswestry Disability Index

**Table 8:** Multiple linear regression analysis of spinal pelvic parameters and JOA

Variables	*B* value	Standard error	*t* value	*P* value	*R*^2^ value
PI	0.192	0.022	8.664	< 0.001	0.687
PT	− 0.119	0.019	− 6.101	< 0.001	
SS	− 0.213	0.020	− 10.754	< 0.001	
Constant	3.043	0.400	7.602	< 0.001	

*PI* pelvic incidence, *PT* pelvic tilt, *SS* sacral slope, *JOA* Japanese Orthopaedic Association score

## Discussion

The multifidus muscle is the most medially located back muscle; it is also the largest muscle, which spans the lumbosacral junction; in addition, it contributes to maintaining the erector posture of the trunk and to rotating and abducting the trunk [9]. Degeneration of soft tissue structures occurs in adult spinal degenerative disease [10]. Consequently, LMA may result in instability of the spine and exacerbate disc and facet degeneration in the lumbar spine [11]. Paraspinal muscle plays a more important role in maintaining the stability of L3–L4 segment than others [12]. This may explain why the apical vertebras of ADS patients most commonly occurred in L3 or L4 segment.

Hypotheses describing the mechanisms of LMA include disuse, denervation, inflammation, and injury [13–16]. In ADS patients, disuse and immobilization of the back muscles are common; these changes may cause atrophy at different intervertebral levels; furthermore, paraspinal denervation and re-innervation are common in disc herniation or nerve root compression [17]. Considering multifidus muscle is innervated by the dorsal root of the lumbar spinal nerve, atrophy of multifidus muscle innervated by medial branch of the dorsal ramus of the lumbar nerve root would occur when the nerve root is compressed by herniated mass [18]. Sun et al. [17] stated that as there was no denervation phenomenon at the L3–L4 level, LMA could be the cause of disc degeneration; at the L5–S1 level, however, LMA could also be the consequence of L4–L5 disc herniation; pathogenetic mechanisms of U-LMA and L-LMA were different. Similarly, our study showed that LMA on the concave side was more severe than that on the convex side; on the convex side, there were differences in the causes of U-LMA and L-LMA. These indicated that the method, in which the axial image obtained at the level of the apex of the curvature was used as a reference for comparison to minimize the effect of the deformity on the morphometry of the paraspinal muscle, in the previous study might not be proper [19]. U-LMA may be the cause of ADS, while L-LMA may be the consequence of ADS. The positional change of the morphometry of paraspinal muscles will be influenced by the difference in the length of the arc in both sides of scoliosis; this is positively related to the radius of the arc; in addition, it is also proportional to their distance from the center of the axis of the spine. The CSA of paraspinal muscles can sometimes show discordant patterns of differences; the possible explanation is that the paraspinal muscles far from the axis of the spinal column will show the obvious effect of the positional change more than others that are closer to the axis of the spine; however, the influence of positional change on multifidus muscles may be caused by the change in the muscle itself, including the atrophy on the concave side or hypertrophy on the convex side [5, 19]. Therefore, patients in supine position during the MRI examination will not influence the evaluation of LMA.

Results of Pearson correlation analysis of spinopelvic parameters and LMA in our study showed that there were correlations among LL, SS, PI, and PT without TK. This is because spinopelvic parameters are geometrically related, such that PI is equal to the sum of SS and PT; variations in the lower arc of lordosis are determined by the sacral slope; when the sacral slope increases, the lower arc of lordosis increases, then the global curvature of lordosis increases as well; thoracic segments are supported by ribs with relatively poor compensatory ability; therefore, the correlation between TK and other radiographic parameters is not significant [20]. Acting like a bowstring, the multifidus muscle could switch compression loading to stretch loading and transmit some of the axial compression force on the disc to the anterior longitudinal ligament, then maintains the spinopelvic parameters [21]. However, our study showed that L-LMA on both concave and convex sides were positively correlated with PT; U-LMA on the concave side was positively correlated with LL; L-LMA on the concave side was positively correlated with PI. These revealed that when the ADS occurred, bowstring effect of multifidus muscle would be influenced by multiple complex factors; the status of multifidus muscle on the concave side would play more important roles in maintaining spinopelvic parameters than others.

A high pelvic incidence is associated with long, curved lumbar lordosis; this reciprocal association is an important component of overall sagittal alignment [22]. It was reported that lumbar hypolordosis was associated with lateral listhesis, vertebral rotation in ADS patients, which would aggravate scoliosis [23]. Therefore, in our study, LL and PT are both protective factors of CA; LL was positively correlated with SVA. PI reflects compensatory ability of the lumbar spine and pelvis in maintaining global alignment of the spine. In patients with high PI, the occurrence of ADS may mean a more severe decompensated state than others. In consequence, PI was positively correlated with CA and CVA in our study [24]. A previous study reported that there was a correlation between an anterior shift in the C7 plumb line and a vertically oriented sacrum [25]. In addition, the sacral slope was positional parameters that can be affected by changes in the alignment of the lower extremities, which would also influence CVA [26]. Therefore, SS was negatively correlated with SVA in our study.

The correlations of LMA at different intervertebral levels and severity of ADS were analyzed in our study. Results showed that U-LMA and L-LMA on the concave side were positively correlated with CA; U-LMA and L-LMA on the convex side were negatively correlated with CA. Yagi et al. [7] suggested that ADS patients did not have an age-related, progressive global muscle weakness, but rather a localized myopathy, which was commonly seen in patients with dropped head syndrome. Significant asymmetric LMA may be the primary cause of ADS and could be used to predict the progression of ADS. Convex U-LMA and concave L-LMA were negatively correlated with the SVA. This indicated that LMA on the diagonal through the apical vertebra would balance the stress and secure the stress conduction via parallelogram effect, which was very important to maintain sagittal imbalance. This parallelogram effect is first proposed in this study. Considering sagittal imbalance has a more disastrous influence on the clinical outcome than coronal imbalance in ADS patients, the significance of CVA may be limited [24]. *R*^2^ value of multiple linear regression analysis of LMA and CVA was very low in our study, which indicated that LMA could not accurately predict CVA. This might be explained by the effects of various factors on CVA [27].

Schwab et al. [28] reported that no significant correlation was found between adult scoliosis and nutritional status or VAS scores in elderly patients. Similarly, our results showed that none of the parameters were correlated with VAS score. Therefore, VAS score would not be an effective method to measure the severity of ADS. In our study, PI was positively correlated with ODI; however, PT and SS were negatively correlated with ODI. Mac-Thiong et al. [29] found that sagittal spinal balance was strongly correlated with ODI in ADS; however, coronal spinal balance did not influence the ODI in their study. Therefore, influencing factors of SVA and ODI may be overlapping. Results in our study showed, at the lower intervertebral level, LMA on the concave side was positively correlated with ODI; in contrast, LMA on the convex side of ODI was negatively correlated with ODI. These indicated LMA at the lower intervertebral level would have a predictive effect on ODI in ADS patients. Compared with ODI, JOA scores are more focused on evaluating neurologic functions [30]. It was reported that worse sagittal spinopelvic alignment was the main cause of functional loss in ADS patients [20]. Therefore, JOA score seemed to be more correlated with spinopelvic parameters than LMA in our study.

There are several limitations associated with our study. First, this is a retrospective cross-sectional study that may result in unavoidable selection bias. Secondly, this is a single-center study and sample size is thus limited. Third, a comparative analysis of different phases of ADS was not conducted as the course ADS is difficult to follow. Studies with a larger sample size that include patients at different stages of disease progression are warranted to confirm the results of the present study.

## Conclusion

In ADS patients, LMA on the concave side is more severe than the convex side; on the convex side, there are differences in the causes of U-LMA and L-LMA. LL and PT are both protective factors of CA progression; high LL is a risk of sagittal imbalance. High SS is a protective factor of sagittal imbalance. Asymmetric LMA may be positively correlated with CA. LMA on the diagonal through the apical vertebra may be very important to maintain sagittal imbalance via parallelogram effect. In addition, LMA at lower intervertebral levels of the apical vertebra may have a predictive effect on ODI. JOA score seems to be more correlated with spinopelvic parameters than LMA.

## Data Availability

Please contact the author for data requests.

## Abbreviations

ADS: Adult degenerative scoliosis
CA: Coronal Cobb angle
CSA: Cross-sectional area
DLS: Degenerative lumbar scoliosis
LL: Lumbar lordosis
L-LMA: Lower intervertebral level LMA
LMA: Lumbar multifidus muscle atrophy
MF: Multifidus
MRI: Magnetic resonance imaging
PI: Pelvic incidence
PS: Psoas
PT: Pelvic tilt
SD: Standard deviations
SS: Sacral slope
SVA: Sagittal vertical axis
TK: Thoracic kyphosis
U-LMA: Upper intervertebral level LMA
